# Growth Simulation and Discrimination of *Botrytis cinerea*, *Rhizopus stolonifer* and *Colletotrichum acutatum* Using Hyperspectral Reflectance Imaging

**DOI:** 10.1371/journal.pone.0143400

**Published:** 2015-12-07

**Authors:** Ye Sun, Xinzhe Gu, Zhenjie Wang, Yangmin Huang, Yingying Wei, Miaomiao Zhang, Kang Tu, Leiqing Pan

**Affiliations:** College of Food Science and Technology, Nanjing Agricultural University, No.1, Weigang Road, Nanjing, Jiangsu, 210095, China; Universita degli Studi di Pisa, ITALY

## Abstract

This research aimed to develop a rapid and nondestructive method to model the growth and discrimination of spoilage fungi, like *Botrytis cinerea*, *Rhizopus stolonifer* and *Colletotrichum acutatum*, based on hyperspectral imaging system (HIS). A hyperspectral imaging system was used to measure the spectral response of fungi inoculated on potato dextrose agar plates and stored at 28°C and 85% RH. The fungi were analyzed every 12 h over two days during growth, and optimal simulation models were built based on HIS parameters. The results showed that the coefficients of determination (R^2^) of simulation models for testing datasets were 0.7223 to 0.9914, and the sum square error (SSE) and root mean square error (RMSE) were in a range of 2.03–53.40×10^−4^ and 0.011–0.756, respectively. The correlation coefficients between the HIS parameters and colony forming units of fungi were high from 0.887 to 0.957. In addition, fungi species was discriminated by partial least squares discrimination analysis (PLSDA), with the classification accuracy of 97.5% for the test dataset at 36 h. The application of this method in real food has been addressed through the analysis of *Botrytis cinerea*, *Rhizopus stolonifer* and *Colletotrichum acutatum* inoculated in peaches, demonstrating that the HIS technique was effective for simulation of fungal infection in real food. This paper supplied a new technique and useful information for further study into modeling the growth of fungi and detecting fruit spoilage caused by fungi based on HIS.

## Introduction

Spoilage fungi usually cause a lot of loss of fruits and vegetables postharvest, and further lead to food safety and security concerns. Diseases in postharvest fruits and vegetables are mainly gray mold, soft rot, and anthracnose caused by *Botrytis cinerea* (*B*.*cinerea*), *Rhizopus stolonifer* (*R*.*stolonifer*), and *Colletotrichum acutatum* (*C*.*acutatum*), respectively. For example, the peach fruits harvest at hot and rainy season, they could be unfavorable for long time storage and transportation, since they are susceptible to spoilt owing to the fungi. *B*.*cinerea* can damage both flowers and fruit [1–2], which causes browning and rotting on the surface of fruit; what’s more, as time goes on, the other around fruit would rot and form a layer of gray fungi. *B*.*cinerea* mostly occurs on fruits such as strawberry, tomato, and grape [3]. *R*.*stolonifer* can be found in the air and soil, and on the surface of many kinds of tools, which inflicts damage to the fruit. Then the sporangium and sporangiospore grow up on infected fruit and diffuse by insect activity and shaking by wind. Subsequently, adjacent healthy fruit will become infected, which is a more common occurrence in strawberry, melon, and peach. When *C*. *acutatum* starts, a small brown circle appears and spreads out over the surface of the fruit, and then becomes deeply brown [4].

In order to reduce economic loss and improve the quality and safety of fruit, further study of the presence or growing status of spoilage fungi in fruit is necessary. Several statistical models have been reported to describe the growth of different micro-organisms related to food safety and quality. Predictive food microbiology encompasses such disciplines as mathematics, microbiology, engineering, and chemistry to develop and apply mathematical models to predict the responses of microorganisms to specified environmental variables [5]. Models were established to describe the mathematical functions between microbe quantity and time [6]. Under the specific culture conditions, the primary model showed that some factors of microorganisms change over time, for example, total viable count, and toxin and metabolite concentrations. Then they used the primary model to describe and fit for delay time, maximum specific growth rate, and microbial growth information [7–8]. For example, Baranyi et al. [9] developed a predictive model about the effect of temperature and water activity on *Aspergillus Niger’s* growth rate to determine the sources of the error when used for prediction. Gougouli and Koutsoumanis [10] simulated the growth of *Penicillium expansum* and *Aspergillus Niger* at constant and fluctuating temperature conditions by measuring the growth rate (*μ*) and apparent lag time (*λ*) of the mycelium growth. However, it was not a facile and rapid method for detecting the total viable count, toxin and metabolite concentrations. Moreover, the process would destroy the samples. These traditional microbiological enumeration techniques in modeling microbial growth were time-consuming and laborious.

Several studies have tried non-destructive techniques like spectroscopy to achieve simple, rapid, and inexpensive methods for the detection of fungal contamination and toxins in cereals [11]. It is generally known that, with the growth of the colony of microorganisms, the color, shape, and chemical compositions of the colony change, affecting the spectral absorption of light interacting with the microbial colony. Other research carried out for fungal identification based on hyperspectral imaging system includes a study by Yao et al. [12], the study focused on the feasibility of using spectral image data for fungal species classification, and the results indicated that all five fungi are highly separable with classification accuracy of 97.7% with only three narrow bands centered at 743, 458 and 541 nm. The application of hyperspectral imaging for food inspection has been investigated by Delwiche et al. [13]. They used hyperspectral reflectance imaging at two selected wavelengths in the near infrared spectrum to separate healthy wheat kernels from those damaged by Fusarium head blight (FHB), a disease caused by the fungus *Fusarium graminearum*. Gómez-Sanchis et al. [14] showed that a hyperspectral reflectance imaging system could detect infections caused by *Penicillium digitatum*in citrus fruits at early time points. Del Fiore et al. [11] used hyperspectral imaging for the early detection of toxigenic fungi on maize. The previous results showed that hyperspectral imaging is able to rapidly discriminate between commercial maize kernels infected with toxigenic fungi and uninfected controls when traditional methods were not yet effective (i.e., from 48 h after inoculation with *A*. *niger* or *A*. *flavus*). Thus, it was possible and feasible to monitor the different growth phases of microorganisms by hyperspectral imaging, as well to distinguish different strains of microorganism.

However, to our knowledge, there have been no studies concerning growth simulation and species discrimination of postharvest fungi by hyperspectral imaging. Thus, this study used three typical spoilage microorganisms which are frequently found in postharvest fruit, such as *B*. *cinerea*, *R*. *stolonifer*, and *C*. *acutatum*. The growing stages of every 12 h over two days (i.e., 0, 12, 24, 36, and 48 h) were measured by a hyperspectral imaging system (HIS). The objectives of the present work were 1) to develop the optimal calibration models for simulating fungal growth based on HIS parameters; 2) to build models to distinguish among three fungi by spectral responses; 3) and to evaluate the feasibility of HIS to simulate and distinguish fungi growth on real food of peaches.

## Materials and Methods

### 1. Fungal strains and culture conditions

*B*. *cinerea* and *R*. s*tolonifer* were bought from Guangdong Micrology Culture Center (Guangdong, China), and *C*. *acutatum* was supplied by College of Food Science and Technology at Nanjing Agricultural University of China. All strains were grown on potato dextrose agar (PDA) plates at 28°C and 85%humidityfor 7 days. Spores were suspended in sterile distilled water containing 0.1% (w/v) Tween 80 and the surface of the medium was washed gently with a sterile pipette. After filtering the spore suspension through four layers of sterile medical tissue, the final spore concentration was determined by hemocytometer and adjusted to 4×10^5^ ascospores/mL.

### 2. Sample preparation and grouping

PDA plates were inoculated with 100 μL ascospore suspension of each fungus, and achieved a final concentration of 40000 CFU for each sample. The plates in control group were inoculated with 100 μL sterile water. After inoculation, the plates were incubated at 28°C with 75% relative humidity in a constant temperature and humidity incubator. There were 660 samples for four groups, and 30 plates for each time points (i.e., 0, 12, 24, 36, 48 h). Since *B*. *cinerea* grew the most slowly among the three fungi, its growing period was adjusted to 72 h in order to achieve the growth curveprofile. Hence, 660 samples were prepared, with 210 for *B*. *cinerea*, 150 for *R*. s*tolonifer*, 150 for *C*. *acutatum*, and 150 for the control.

The “Xiahui 5” peach samples were handpicked from orchard of academy of agricultural sciences of Jiangsu province. The used peaches without any defects, bruises, diseases and contamination were selected at a maturity of 70% and then divided into four groups. There were 30 peaches in every group at each detection time. 1% sodium hypochlorous acid liquid was used to sterilize for 2 minutes and then repeatedly cleansed them using sterile distilled water. After the peaches dried by airing at room temperature, they were inoculated with ascospore suspension of each fungus, and achieved a final concentration of 40000 CFU for each peach. The samples were placed orderly in the thermotank and stored at 20°C of shelf temperature. At each 12 hours of the experiment, 30 samples were withdrawn randomly for hyperspectral imaging and referenced microbiological analysis. When the peaches severely rotted, the experiment was stopped.

### 3. Hyperspectral imaging system

**Fig 1:**
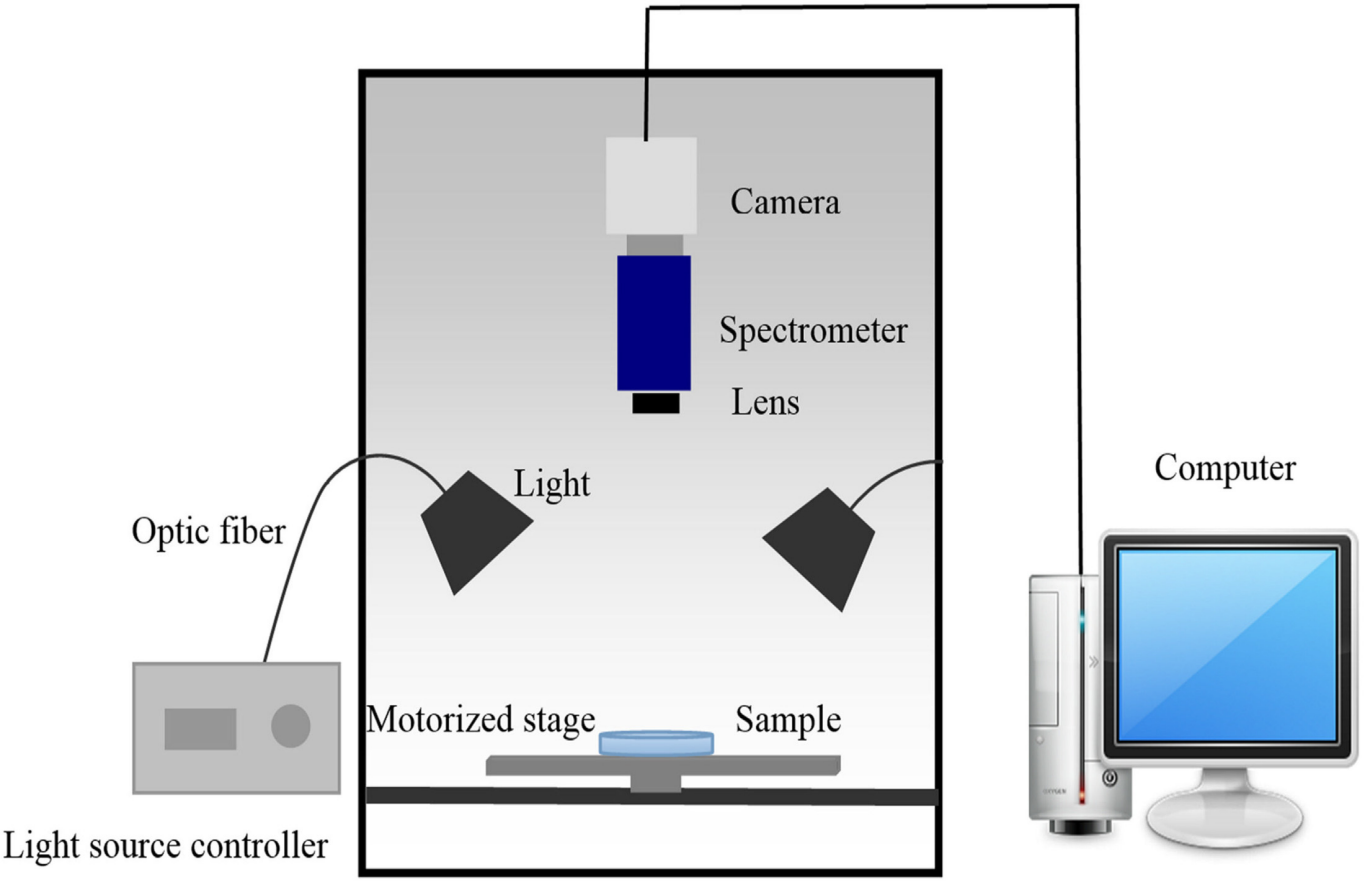
The schematic diagram of hyperspectral imaging system.

Hyperspectral images of fungi on plates were acquired using a lab-scale hyperspectral imaging system in reflection mode (Fig 1). The system mainly consisted of a CCD camera (ICL-B1620, Imperx, USA), an imaging spectrometer (ImSpectorV10E, Specim, Finland), a variable-focal-length lens, tunable light source (a 150 W halogen tungsten lamp controller, 3900ER, Illumination Technologies Inc, USA), horizontal motorized stage (IRCP0076 of-ICOMB001, Isuzu, Taiwan, China), a computer with the image acquisition software (Spectral Image, Isuzu, Taiwan, China). The effective wavelength of the imaging system was 400 to 1000 nm.

### 4. Hyperspectral image acquisition

In order to acquire accurate data, parameters of the hyperspectral imaging system needed to be adjusted before image acquisition [15]. After preliminary experiments, the parameters were set as follows: the distances of the camera and the light source from the target were 30 cm and 20.5 cm, respectively; and the conveyor speed was adjusted to 2.5 mm/s. For the detection of plates, the intensity of line light source was 67.5W, at a 45°angle to the sample; the exposure time was 4 ms. For the detection of peaches, the intensity of line light source was 52.5W, the exposure time was 5 ms.

The spectra are composed of 420 wavelengths over the spectral range from 400 to 1000 nm. The spectral and spatial resolution of hyperpctral images are 1.43 nm and 0.205 mm, respectively. Spectral measurements of fungal mycelia grown on PDA were acquired without the dish cover.

Due to the presence of the dark current in the camera and the impact of external factors, images contain noise [16]. The hyperspectral images were corrected with a white and a dark reference images, implement by the image acquisition software [17]. The dark correction can subtract the background noise, and the white correction can calibrate the variations in the light source. Covering the camera lens with its opaque cap completely provided the dark reflection image (R_d_), and a Teflon white board (99.99% reflectivity) was used to obtain the white reflection image (R_t_) [18]. The corrected relative image R was calculated according to the following equation:

$$R=\frac{R_{0}-R_{d}}{R_{t}-R_{d}}$$ (1)

Where R_o_ was the original hyperspectral image; R_d_ is the dark image and R_t_ was the white reflectance image. The corrected images were used to extract spectral information, select effective wavelengths, build the optimal calibration model, and distinguish among different fungal species.

### 5. Colony count

After the hyperspectral images acquisition, the total colony count on the plates and peaches were calculated with the official analysis method issued in 2010 in China (GB 2010–4789.15) [19].

### 6. Hyperspectral imaging processing

At first, the regions of interest (ROI) were obtained using ENVI software (ENVI4.7, Research System Inc., Boulder, CO, USA). The locations where fungal mycelia grew were selected and an ROI with 2500-pixel was selected. If there was no fungal mycelium on the plate, ROIs were selected in the middle of the plate. Then mean spectral value of the ROI from the hyperspectral images was calculated. In total, 660 mean values were obtained from ROIs of all plates. For the peach simples, the ROIs were selected at the location of inoculation, the other processing methods were the same as the plates.

### 7. Data analysis and fitting model development

In this work, in order to fit the fungi growth, three methods were used to extract characteristic information from the original spectra without any preprocessing. Thus, 660 data points in total from four groups were collected, and each fungus had 150 data points (210 data points for *B*.*cinerea*). The average value of 20 data for each time point was used to simulate the fungi growth, then the other 10 data points were used to test the model. The following three methods process the full spectral into one value for each time point, thus each time point has only two spectral values for calibration and testing, respectively. The first method was by using the average value of spectral response in the full wavelengths of 400–1000 nm, which can compress the complex multivariable data down to a single datum (Method I). The second method relied on choosing the value of the wavecrest for analysis and modeling (Method II), because the spectral values of fungi have the maximum difference at the wave crest, and the wave crest is easy to obtain. Principal component analysis (PCA) is widely used for dimensionality reduction and feature extraction [20–21]. So the third method was by analyzing all bands of 400–1000 nm based on principal component analysis (Method III) with SPSS 18.0 software (The SPSS Inc. Chicago., USA). We used the score of first principal component for simulation. This characteristic information was used to set up a growth curve using the statistical toolbox of Matlab 7.1 (The Math Works Inc. Natick, USA). For the application of HIS in real food, we used the above methods to fit the fungi growth in the peach samples too.

The ANOVA was performed to demonstrate that whether HIS can recognize the surface alterations due to the postharvest pathogen. Duncan’s multiple comparison tests was used to determine the differences between the means of spectral response values. The means are acquired from Method II. Statistical analyses were carried out using SPSS 18.0 software.

For the analysis of the fungal species, PCA was performed for all spectra values of three kinds of fungi and control group at each time point. The PC scores were used to plot a model for different fungi identification for each time point.

In order to further distinguish between species, the partial least squares discrimination analysis (PLSDA) in PLS Toolbox 7.5 (Eigenvector Research, Inc., Wenatchee, Wash., USA) was used to build a discrimination model. Before the modeling, the spectra were preprocessing by the function of 'autoscale', included in the PLS Toolbox 7.5. The 20 spectral values for each specie and time point were used as the calibration set and the remaining 10 samples were used for testing. The coefficients of determination for calibration (R_c_^2^) and testing (R_t_^2^), the sum square error (SSE), and root mean squared error (RMSE) were compared to determine the best simulation models.

The correlation coefficients between the growth curve fitting by hyperspectral imaging parameters and the growth curve fitting by the traditional techniques of colony forming units were used to evaluate the performance of simulation models.

## Results and Discussion

### 1. The growth of fungi at different culture stages

**Fig 2:**
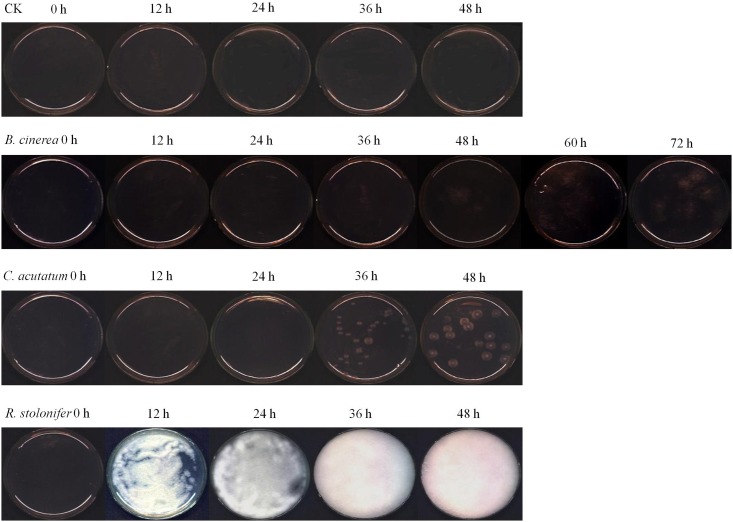
Typical hyperspectral RGB images of different fungi (R: 662 nm, G 554.5 nm, and B 450 nm).

Fig 2 showed the RGB (red for 662 nm, green for 554.5 nm, and blue for 450 nm) images of fungi inoculated on plates after storage at 28°C and 85% RH for 0, 12, 24, 36, 48, 60 and 72 h, which were taken from the hyperspectral imaging system. The images indicated that *R*. *stolonifer a* growled in fastest rate among the three fungi. At 24 h, the plate was almost full of *R*. *stolonifer*, and completely filled at 36 h and 48 h. The growth of *B*. *cinerea* was the slowest, and it was hard to see mycelium before 36 h. The colony morphology of *C*. *acutatum* was circular, smooth and white, and it was difficult to observe mycelium before 24h.

### 2. Spectral characteristics of fungi

**Fig 3:**
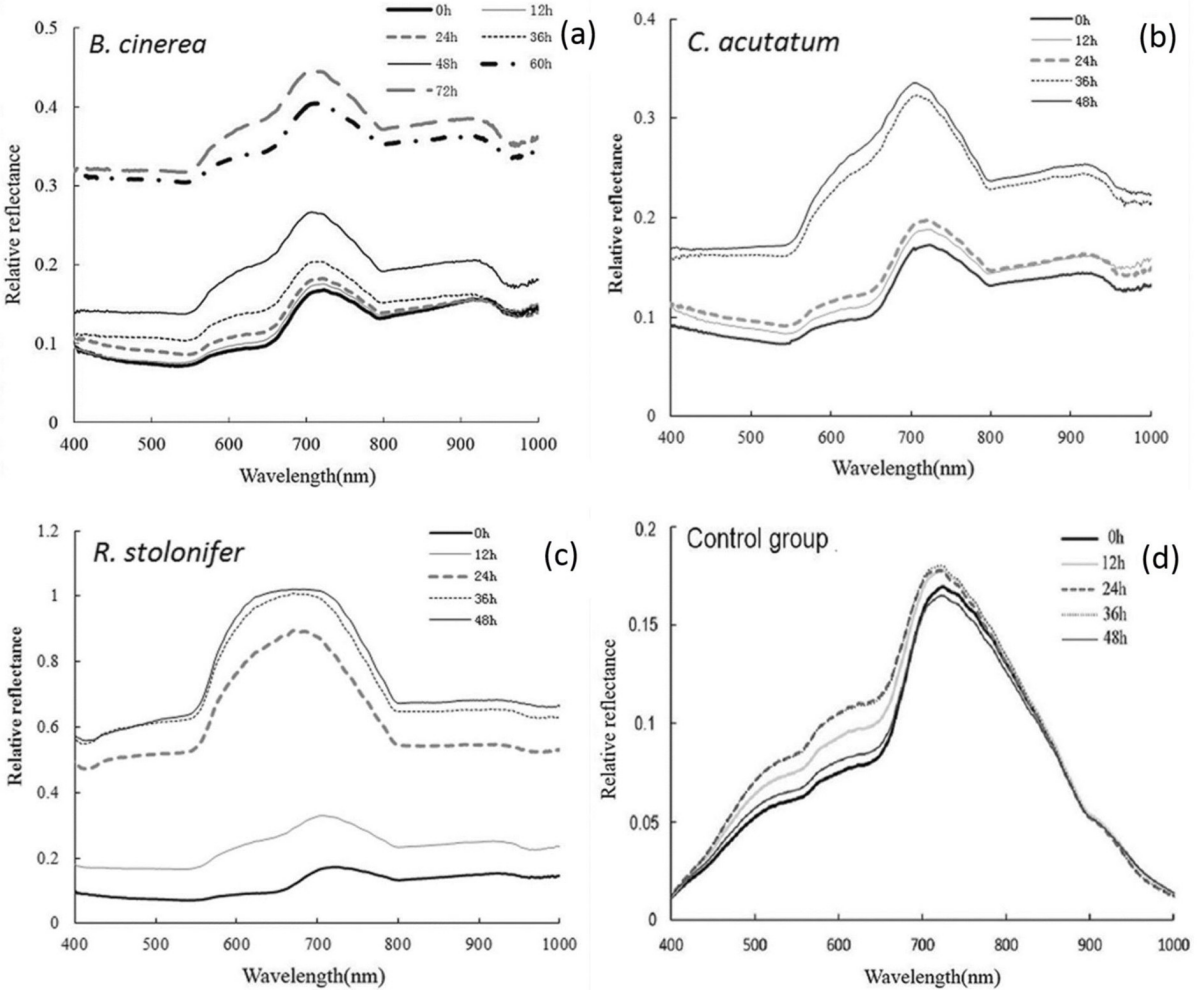
Average original spectra of fungi.

The typical reflectance spectra of *B*. *cinerea*, *C*. *acutatum*, *R*. *stolonifer* and control group at different culture times were shown in Fig 3a–3d. The mean spectra were calculated based on pixels of the ROIs from all 30 samples. The resulting 2500 pixel spectra, each with 420 data points, can be thought of as a fingerprint, which can be used to characterize the fungi compositional change and metabolic products ascribing to the growing fungi inoculated in the plates.

In this research, the hyperspectral imaging employed wavelength range from 400 to 1000 nm, covering visible wavelength (400–750 nm) and short-wave near-infrared (NIR) (750–1000 nm). As shown in Fig 3, in the same condition, the control group changed slightly, while the other three spectral values changed a lot. So the HIS allow the recognition of surface alterations due specifically to the postharvest pathogen. For three fungi, reflectance spectra values increased with culture time, and the biggest difference of relative reflectance was found at the wave peak around 716 nm in different fungi. The results are not accordance with the findings on the fungi infection in food. Generally speaking, spectral reflectance signals decreased with increasing levels of fungal infection [11, 22]. This is due to the fungi growth of plate culture was different from that growth and reproduces in food materials. This phenomenon was mainly attributed to the growth of *B*. c*inerea*, *C*. *acutatum* and *R*. *stolonifera* which produced white mycelia during the early culture time (0–48 h), including the synthesis of chemical composition of mycelia, metabolic products and the degradation of nutrients in culture medium. With culture time extension, the white mycelia became more obvious and had a reflection of greater intensity. As a result, the fungi had weak spectral absorption over the spectral region of 400–1000 nm. Therefore, before the colonies generated the other color, the reflection spectral value increased with time, and it is very noticeable that the reflectance value at 48 h (or 72 h for *B*. c*inerea*) was the highest.

As shown in Fig 3, three conspicuous absorption peaks was located at about 560 nm, 770 nm and 970 nm. The first two absorption peaks were identified as the region of visible spectrum, the absorption peak of 560 nm was associated with the absorption of green light and the molecular chemical bonds related to benzene rings, and 770 nm was associated with the absorption of red light. As we know that, green and red were the complementary color, so possibly when they were mixed in a certain proportion, the white hypha was close to white. These results were in accordance with those published by Fernández-Ibañezetal [23], who studied a rapid detection of aflatoxin B1 in maize and barley, demonstrated that spectral differences in visible wavelength were associated with bands ascribed to color changes of fungal. During the NIR wavelength (850–1000 nm), changes in reflectance spectra were due to the light scattering caused by the fungal growth on the surface. The last absorption peak observed close to 970 nm was mainly ascribed to the second overtone O–H stretching from water due to the fact that water was the major component in the mycelium [24–26].

From Fig 3a, the spectral response of *B*. *cinerea* showed a wide gap between 48 h and 60 h during growth, the change of spectral was consistent with the growth change of fungi (Fig 2). The reason was that the colony grew quickly in this period and reached the logarithmic phase. Spectral response values of *C*. *acutatum* (Fig 3b) and *R*. *stolonifer* (Fig 3c) changed rapidly in the growing stage from 24 h to 36 h, and 12 h to 24 h, respectively. They were also consistent with the growth change of fungi. It meant that the spectral values reflected the fungal growing status, and can further reflect the difference between the growth stages; it could be advantageous to use spectral reflectance values to establish growth curves related to the culture time. This pattern was also described in the study of fungal infected on brown rice conducted by Siripatrawan and Makino [27], who reported that the HIS spectral signals reflect the increasing phase of colony counts, what’s more, HSI technique was effective for prediction the colony counts of fungal infection in rice grains.

Table 1 showed the results of variance analysis of wave crest value for plates inoculated with different fungi during storage. Statistical analyses revealed that it was significantly different among the groups. They didn’t differ significantly among the different time points for control group at a level of *P* = 0.01. Among three fungi, took *B*. *cinerea* as an example, the values on the growth of 0, 12 and 24 h were the letter of ‘e’. However, they had the different letters on 36, 48, 60 and 72 h. The results were identical to that described in Fig 2. It was obvious that the images of control group had no significant differences. Similarly, the pictures of *B*. *cinerea* at 0, 12 and 24 h looked the same. With the growth of mycelium, the spectral values obviously increased in logarithmic growth phase as showed in Fig 3a,. The similar results were found for *C*. *acutatum* and *R*. *stoloifenr*. Therefore, the results proved that the change of spectrum were due to the mycelium growth. It demonstrated that HIS can recognize the surface alterations due to the postharvest pathogen.

**Table 1:** Variance analysis of wave crest value of for control and three fungi at different growth stage.

Time point (h)	Spectral values of wave crest			
	*B*.*cinerea*	*C*. *acutatum*	*R*. *stoloifenr*	Control group
**0**	641.4±40^e^	695.9±38.2^c^	686.9±37.9^e^	682.8±20.2^a^
**12**	690.6±71.7^e^	786.6±75.1^b^	1287.2±92.3^d^	710.5±24.8^a^
**24**	725.3±58.6^d^ ^e^	782.2±66.5^b^	3316.2±176.9^c^	710.8±60.9^a^
**36**	829.2±41.1^d^	1290±100.1^a^	3815.3±188.2^b^	720.1±25.1^a^
**48**	1048.5±130.3^c^	1357±151.6^a^	3927.9±133.7^a^	679.4±21.5^a^
**60**	2497.1±340.3^b^			
**72**	2755.5±186.1^a^			

^a-e^ Means in a column followed by a different letter differ significantly at *P* = 0.01 by Duncan’s multiple range tests. Data were means ± SD of thirty replicates.

### 3. Growth simulation of fungi based on the colony forming unit (CFU)

**Fig 4:**
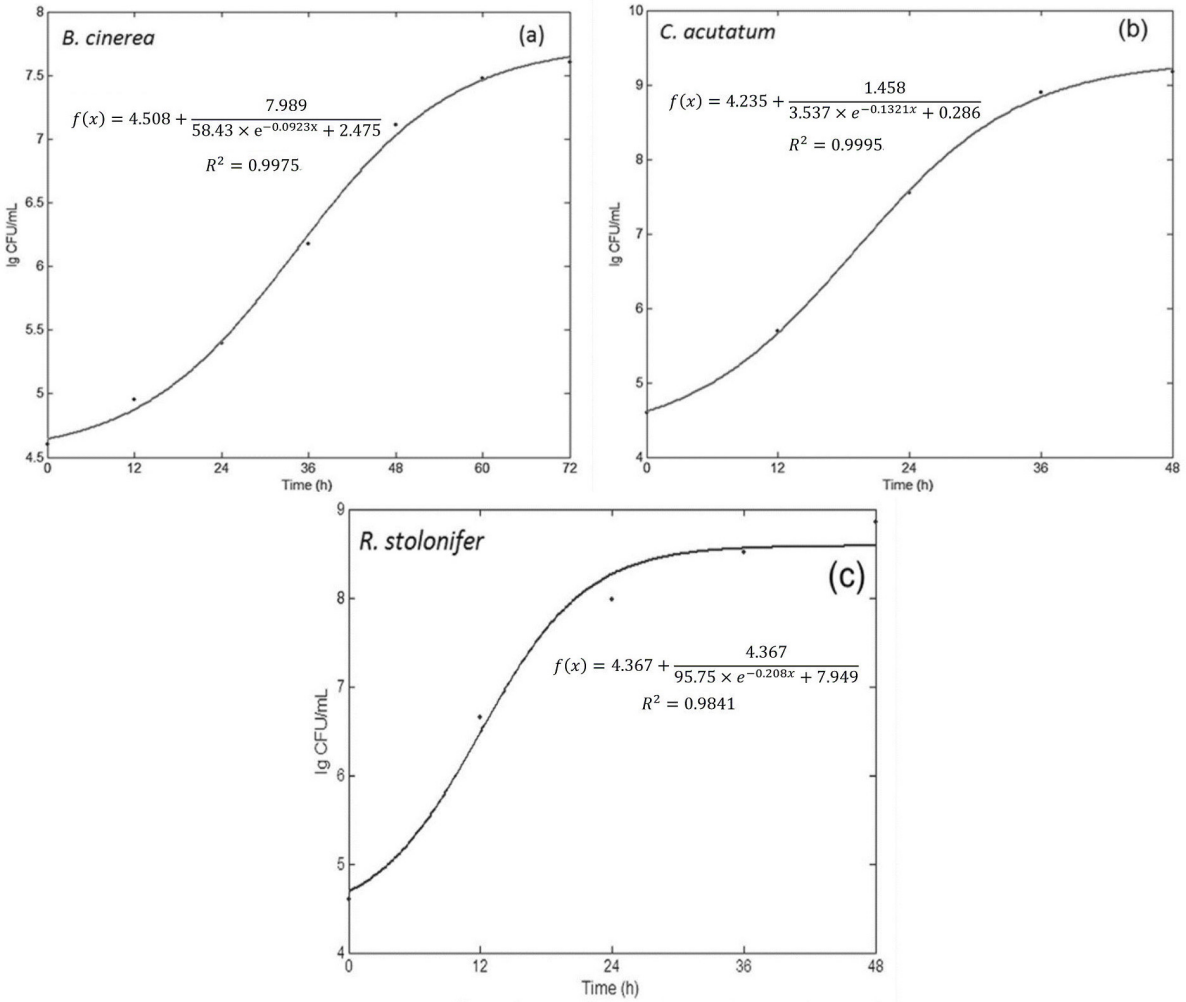
Fitting the growth curve of colony forming unit-culture time by MATLAB.

The total numbers of fungi on the plates were calculated using the official analysis method for food issued in China. The curve was simulated by a logarithmic function of CFU, as shown in Fig 4a–4c. The CFU of *B*. *cinerea* on the seven stages of growth at 0, 12, 24, 36, 48, 60 and 72 h were obtained. The CFU of *B*. *cinerea* were 4.0×10^4^, 9.0×10^4^, 2.5×10^5^, 1.5×10^6^, 1.3×10^7^, 3.0×10^7^ and 4.0×10^7^, respectively, and the logarithm (base 10) were 4.60, 4.95, 5.40, 6.18, 7.11, 7.48 and 7.6. Thus, the simulation equation was acquired, and the growth curve was shown in Fig 4a, with a coefficient of determination (R^2^) of 0.9975. For the *C*. *acutatum*, the colony forming unit (CFU) of on the five stages of growth at 0, 12, 24, 36 and 48 h were 4×10^4^, 5×10^5^, 3.5×10^7^, 8×10^8^ and 1.5×10^9^, respectively. The logarithms (base 10) of the values were 4.6, 5.69, 7.54, 8.9 and 9.17. Then the equations of growth simulation based on CFU were shown in Fig 4b, the R^2^ of the model reached 0.9995. The CFU of *R*. *stolonifer* for 0, 12, 24, 36 and 48 h were 4×10^4^, 4.5×10^6^, 9.5×10^7^, 3.3×10^8^ and 7×10^8^, respectively, and the logarithms (base 10) of the values were 4.6, 6.65, 7.98, 8.51 and 8.85, respectively. The equation of growth simulation was displayed in Fig 4c, the R^2^ was 0.9841. This is the most basic and typical method to simulate the microbial growth. Mold counts in the inoculated plates were not notably different during the first 12 h of incubation for *B*. *cinerea* and *C*. *acutatum*; then increased rapidly after 24 h and12 h for respective *B*. *cinerea* and *C*. *acutatum*; reached a maximum CFU/g after 60 h, 36 h and 36 h of incubation for *B*. *cinerea*, *C*. *acutatum* and *R*. *stolonifer*, respectively. Typically, fungi grew rapidly during the exponential phase, followed by a stationary phase plateau [28]. However, according to the growth curve of Fig 4, the distinction of lag phase, exponential phase and stationary phase was not obvious, because the measurement of culture time is scattered in some degree.

### 4 Growth simulation of three fungi by HIS

In this work, three methods—the average of full spectrum in the range of 400–1000 nm, the response value at wave crest of 716 nm, and the value of principal component score of the full spectrum—were used to extract the characteristic information for the growth simulation of three fungi as shown in Table 2.

**Table 2:** Results of exponential models for three fungi growth of *R*. *stoloifenr*, *B*. *cinerea* and C. acutatum*.

Fungal species	Method	Simulation equation	Calibration dataset			Testing dataset			*r*
			R_c_^2^	SSE(×10^−4^)	RMSE	R_p_^2^	SSE(×10^−4^)	RMSE	
***B*. *cinerea***	I	$f(x)=0.124+\frac{1}{49603.352*e^{-0.18x}+6.81}$	0.9959	0.99	0.007	0.7223	53.40	0.023	0.898
	II	$f(x)=0.178+\frac{1}{77800*e^{-0.195x}+3.92}$	0.9948	3.79	0.011	0.7910	3.34	0.057	0.941
	III	$f(x)=-\text{0}.\text{685}+\frac{1}{141.1*e^{-0.141x}+0.265}$	0.9979	334.00	0.106	0.7897	4.65	0.254	0.932
***C*. *acutatum***	I	$f(x)=0.124+\frac{0.064}{9070*e^{-0.325x}+0.578}$	0.9903	1.67	0.025	0.9367	8.77	0.756	0.899
	II	$f(x)=0.178+\frac{0.092}{9096*e^{-0.326x}+0.589}$	0.9939	1.48	0.014	0.9368	7.74	0.044	0.900
	III	$f(x)=-0.879+\frac{1}{604.3*e^{-0.219x}+0.463}$	0.9813	682.00	0.261	0.8687	14.80	0.504	0.887
***R*. *stoloifenr***	I	$f(x)=0.111+\frac{1}{117.55\times e^{-0.237x}+1.589}$	0.9996	1.23	0.013	0.9815	3.76	0.019	0.954
	II	$f(x)=0.158+\frac{1}{80.15*e^{-0.236x}+1.202}$	0.9960	2.48	0.016	0.9914	2.03	0.011	0.957
	III	$f(x)=-1.443+\frac{1}{27.74\times e^{-0.232x}+0.415}$	0.9996	18.20	0.043	0.9906	3.32	0.129	0.955

*I, II and III represented the different methods I, the average of full spectrum in the range of 400–1000 nm; II, the response value at wave crest of 716 nm; III, the value of principal component score of the full spectrum. R_c_^2^ and R_p_^2^ were the coefficient of determination of calibration and prediction, respectively; SSE and RSE, sum of squares due to error and root mean squared error, respectively; r, the correlation coefficient of prediction accuracy between spectral values and colony forming unit.

#### 4.1 Growth simulation of *B*. *cinerea*

Using method I, the simulation equation, R^2^, SSE and RMSE were showed in Table 2, and with 0.9959, 0.99×10^−4^, 0.007 and 0.7223, 53.40×10^−4^, 0.023 for calibration and testing dataset respectively. Based on the Method II, the calibration model showed a good fit to *B*. *cinerea* growth with high R_c_^2^ of 0.9948, low SSE of 3.79×10^−4^ and RMSE of 0.011. Then the model was validated by the testing dataset, the simulation performance decreased, with a little lower R_p_^2^of 0.7910 and higher RMSE of 0.057. Method III using the principal component scores of all spectral band of 400–1000 nm. Principal component analysis (PCA) showed that the first three principal components accounted for 90.2%, 6.9% and 0.9% of the total variance, respectively. In this work, the score of first principal component (PC1) was selected to simulate the growth of *B*. *cinerea*. The PC1 score on the seven stages were -0.789, -0.598, -0.372, 0.123, 1.692, 2.701 and 3.003. The results in Table 2 showed that the model for the calibration dataset fit well with the growth of *B*. *cinerea*, with 0.9979, 3.34×10^−2^, 0.106 for R_c_^2^, SSE and RMSE, respectively. However, when evaluated against the test dataset, results showed that it didn’t provide the same accurate simulation, with a lower R_p_^2^ of 0.7897, SSE of 4.65×10^−4^ and RMSE of 0.254.

From the results above, the three methods provided a good simulation of *B*. *cinerea* growth (Table 2), though the model using the peak value gave the best simulation accuracy, with the highest coefficients of determination (R_p_^2^ = 0.7910) and the lowest sum square error (SSE = 3.34×10^−4^) in the test dataset. According to correlation between spectral values and colony forming units (CFU) of *B*. *cinerea*, the correlation coefficients (r) were 0.898, 0.941 and 0.932 for the methods of the average of full wavelengths, peak value, and PC1 score, respectively. The peak value showed the best simulation accuracy for the growth of *B*. *cinerea*.

#### 4.2 Growth simulation of *C*. *acutatum*

The three methods were also used to extract the characteristic information for the growth simulation of *C*. *acutatum*, and the model parameters were shown in Table 2. For method I, the model gave a very satisfactory simulation of growth for the calibration, with the R_c_^2^ of 0.9903, SSE of 1.67×10^4^ and RMSE of 0.025, respectively. After validation using the test dataset, the R_p_^2^, SSE and RMSE of the simulation model were 0.9367, 8.77×10^−4^ and 0.756, respectively (Table 2). As found in Table 2 for method II, the R^2^s, SSEs, and RMSEs of the model were 0.9939 and 0.9368, 1.48×10^−4^ and 7.74×10^−4^, 0.014 and 0.044, for calibration and testing dataset, respectively.

The last method used to build the simulation model of *C*. *acutatum* was the score of principal component of the spectral response in full wavelength of 400–1000 nm. Principal component analysis (PCA) showed that the first three principal components accounted for 97.8%, 1.1% and 0.4% of the total variance, respectively. The first principal component (PC1) accounted for the largest variance, so the score of PC1 was used to set up growth simulation model of *C*. *acutatum*. The PC1 score on the five stages of 0, 12, 24, 36 and 48 h were -0.877, -0.856, -0.602, 0.569 and 1.207, respectively. For the calibration dataset, the model also showed good performance with R_c_^2^ of 0.9813, SSE of 6.82×10^−2^ and RMSE of 0.261. For the testing dataset, R_p_^2^, SSE and RMSE were respectively 0.8687, 1.48×10^−3^ and 0.504.

Using the three models for the growth simulation of *C*. *acutatum*, they all presented very good simulation accuracy (Table 2). Furthermore, the model based on the peak value in the full wavelength of 400–1000 nm had best simulation accuracy with highest R^2^s of 0.9939 and 0.9368, lowest SSEs of 1.48×10^−4^ and 7.7.4×10^−4^, RMSEs of 0.014 and 0.044, for calibration and testing dataset, than the average spectral response of full waveband and the score of PC1. Additionally, the correlation coefficient between the spectral response and the colony forming unit (CFU) of *C*. *acutatum* reached 0.900 using the method of the peak value at 716 nm, which was used to set up the simulation model.

#### 4.3 Growth simulation of *R*. *stolonifer*

For method I, the parameters of simulation equation for calibration and testing dataset were 0.9996 and 0.9815 for R^2^, 1.23×10^−4^ and 3.76×10^−4^ for SSE, 0.013 and 0.019 for RMSE, respectively. The R^2^s, SSEs and RMSEs for calibration and testing dataset were respectively 0.9960 and 0.9914, 2.48×10^−4^ and 2.03×10^−4^, 0.016 and 0.011for method II. For method III, principal component analysis (PCA) showed that the first three principal components accounted for 99.7%, 0.2% and 0.05% of the all variance, respectively. So the score of first principal component (PC1) was used to build the growth simulation of *R*. s*tolonifer*. The PC1 score on the five stages were -1.406, -0.979, 0.476, 0.894 and 0.989. The results regarding the model were showed in Table 2. The R^2^s, SSEs and RMSEs for calibration and testing dataset were respectively 0.9996 and 0.9906, 1.82×10^−3^ and 3.32×10^−4^, 0.043 and 0.129.

From the above results, the models presented very high accuracy for growth simulation of *R*. *stolonifer* using the three methods, which include the average spectral response of full spectra, the wave crest response at 716 nm, and the score of PC1. Among them, the model based on the wave crest response at 716 nm gave the best performance, with the highest R_p_^2^, the lowest SSE and RMSE with 0.9914, 2.03×10^−4^, and 0.011, respectively. Furthermore, the correlation coefficient (r) between spectral response and CFU were very high with 0.954, 0.957, and 0.955 for the three methods, respectively. The correlation between spectral response of wave crest at 716 nm and colony forming units (CFU) was highest with r of 0.957.

#### 4.4 Comparisons among three fitting methods

**Fig 5:**
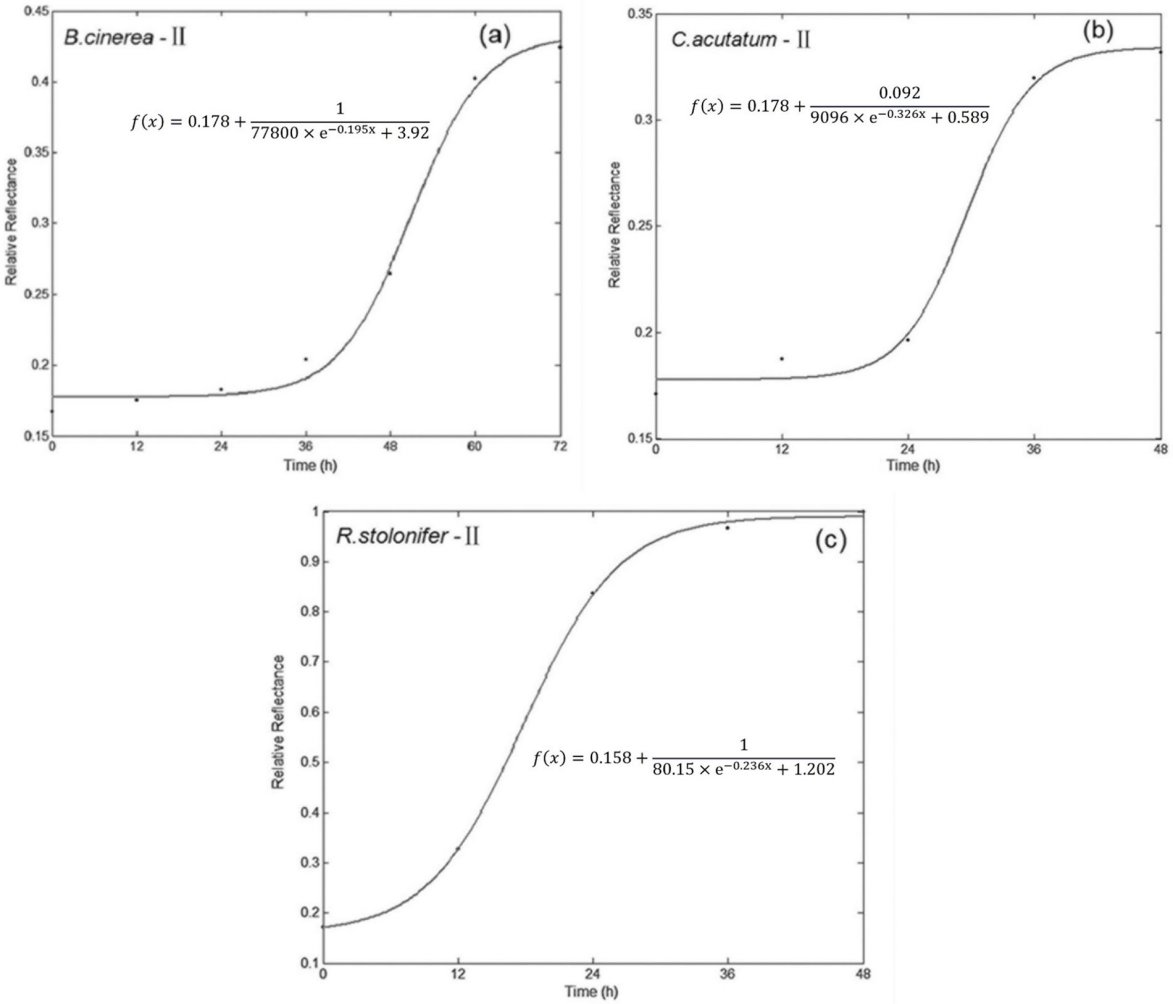
Fitting the growth curve of fungi by Matlab (II: the response value at wave crest of 716 nm).

Fig 5 showed the growth models for three fungi of method II, which gave best fitting accuracy. Other fitting curve figures based on method II and method III for fungi growth could be found in attached supporting information. All of the curves here had a lag phase, logarithmic phase, and stationary phase, however, there was not a clear distinction between the three phases, because of the measurement of culture time was too scattered. Table 2 showed the R^2^, the sum of squares due to error (SSE), and root mean squared error (RMSE) of the models for both calibration and testing datasets. It can be seen that the R^2^s of growth simulation models for the three fungi were 0.9813–0.9996 and 0.7223–0.9914 in calibration and test datasets, respectively, and the SSEs and RMSEs were close to zero. These results indicated that these models can fit well with the fungal growth using methods based on spectral response in the range of 400–1000 nm. Through the R^2^ of validation dataset, the prediction performance of these models for fungal growth can be evaluated. By modeling the growth curve with CFU and culture time, the growth of fungi appeared as S-curves, and the shape was the same as that based on spectral response of fungi. As Table 2 showed, in the test dataset, the second method built using the wave crest at 716 nm had an overall better R_p_^2^ and correlation coefficient between spectral values and CFU, which was also in concurrence with the work reported by He et al. [29], who used several optimal wavelengths to establish models. In addition, it was the easiest among the three methods to process and model, because of using only a single parameter. From another point of view, 716 nm was in the region of visible spectrum, the reasons were allied to the variations of fungal color and texture during culture time [30].

### 5. Analysis of fungal species by PCA

**Fig 6:**
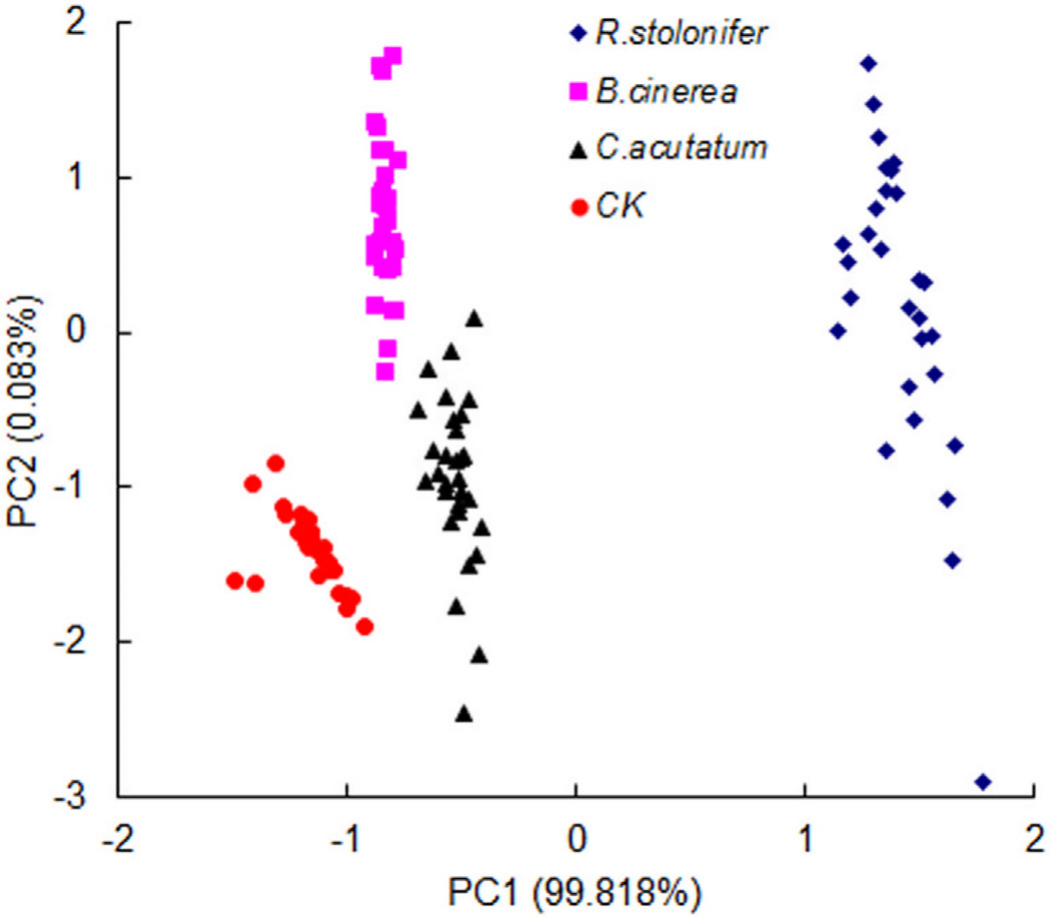
Results of PCA for different fungi at 36 h after inoculation.

The statistical analysis performed using PCA on the full spectral ranges (400–1000 nm) showed the ability to discriminate among the three fungi and control group at four growth stages other than 0 h. The results showed that *R*. *stolonifer* differed highly from the other two fungi and control group at four time points, since the *R*. *stolonifer* developed more rapidly. The data points of control, *B*. *cinerea*, and *C*. *acutatum* overlapped on the growth stages of 12, 24, and 48 h. However, the four groups including *R*. *stolonifer*, *B*. *cinerea*, *C*. *acutatum* and control can be clearly distinguished at the time point of 36 h (Fig 6). Thus, this time point can be used to discriminate among the three fungi based on spectral response. *B*. *cinerea* and *C*. *acutatum* could be discriminated from each other after inoculated for 36 h. To some extent, this was due to the low growing speed and similar surface color in *C*. *acutatum* and *B*. *cinerea*. In the full wavelength range, the spectral absorption of fungi was increased with colony’s growth, and the spectral differences were determined by “scattering” phenomena, the dispersion of light which occurs as a result of the changes produced by fungal growth on the surface of plates [31].

### 6. Discrimination of fungi species by PLS-DA

According to the PCA results, it was possible to discriminate the three fungi at the culture time of 36 h by PLS-DA. The results of discriminant analysis on the full wavelength ranges were showed in Table 3. The discrimination accuracy among *B*. *cinerea*, *C*. *acutatum*, *R*. *stolonifer* and control group for the test set were 100%, 100%, 100% and 90%, respectively. The average accuracy of classification for the test set was 97.5% among the control and three fungi. The results were in line with the PCA results. The misclassification rate of *R*. *stolonifer* in the modeling and test dataset are lowest, because the growth rate of *R*. *stolonifer* was the highest. It had obvious differences with the other two kinds of fungi. In order to obtain more exact and faster discrimination, it is important to find optimal wavelengths in a future experiment. Overall, the spectral analysis enabled discrimination between fungal species. In the future, we can establish a larger number of growth models for strains, and these could be adopted to identify unknown fungal species and growth stages on the basis of their reflectance values of spectra, by comparison with the standard spectral model of known fungi.

**Table 3:** Results of PLSDA models for fungi which cultured for at 36 h.

Sample class	Fungal species	Discriminant result				Recognition rate (%)
		*R*. *stolonifer*	*B*. *cinerea*	*C*. *acutatum*	CK	
**Modelling dataset**	*B*. *cinerea*	0	18	1	1	90
	*C*. *acutatum*	0	0	20	0	100
	*R*. *stolonifer*	20	0	0	0	100
	CK	0	1	0	19	95
	subtotal	20	19	21	20	96.3
**Testing dataset**	*B*. *cinerea*	0	10	0	0	100
	*C*. *acutatum*	0	0	10	0	100
	*R*. *stolonifer*	10	0	0	0	100
	CK	0	1	0	9	90
	subtotal	10	11	10	9	97.5

### 7. HSI application to peach samples

After inoculation of peaches, the color of pathogenic position became deeper, the absorption of light increased, so the spectral reflection value decreased with the detection time. At the storage temperature of 20°C, the peaches had been badly decomposed after 132 h with twelve detection, and lost the edible value, so we stopped the detection of peaches. From the result of section 3.3, method II was the optimal simulating model, therefore we used method II to fit the fungi growth in the peach samples. In this experiment, the spectral value had a decreased trend; however, the colony growth curve presented an increasing trend. In order to understand easily, we used negative spectral values to build the model. The results of fitting model based on hyperspectral imaging system (HIS model) and colony forming unit (CFU model) were showed in Table 4. The three models provided a good simulation for calibration set; the prediction effects were relatively lower. However, HIS model and CFU model had high correlations with 0.941–0.983.

**Table 4:** Results of models for three fungi growth of *R*. *stoloifenr*, *B*. *cinerea* and *C*. *acutatum* in the peach samples*.

Fungal species	HIS Simulation equation	R_c_^2^	R_p_^2^	CFU Simulation equation	r
***B*. *cinerea***	$f\left(x\right)=-0.898e^{-{\left(\left(x-25.07\right)/277.2\right)}^{2}}$	0.9292	0.8594	*f*(*x*) = (2.2*x* ^2^+130.2*x*)10^−4^+2.85	0.954
***C*. *acutatum***	$f\left(x\right)=-0.9005e^{-{\left((x-12.59)/530.2\right)}^{2}}$	0.9835	0.7591	*f*(*x*) = (1.01*x* ^2^+302.7*x*)10^−4^+2.615	0.941
***R*. *stoloifenr***	$f\left(x\right)=-0.9e^{-{\left(\left(x-12.59\right)/464.7\right)}^{2}}$	0.9927	0.7382	*F*(*x*) = (2.9*x* ^2^+22*x*)10^−4^+3.13	0.983

*R_c_^2^ and R_p_^2^ were the coefficient of determination of calibration and prediction, respectively; r, the correlation coefficient of prediction accuracy between spectral values and colony forming unit.

**Fig 7:**
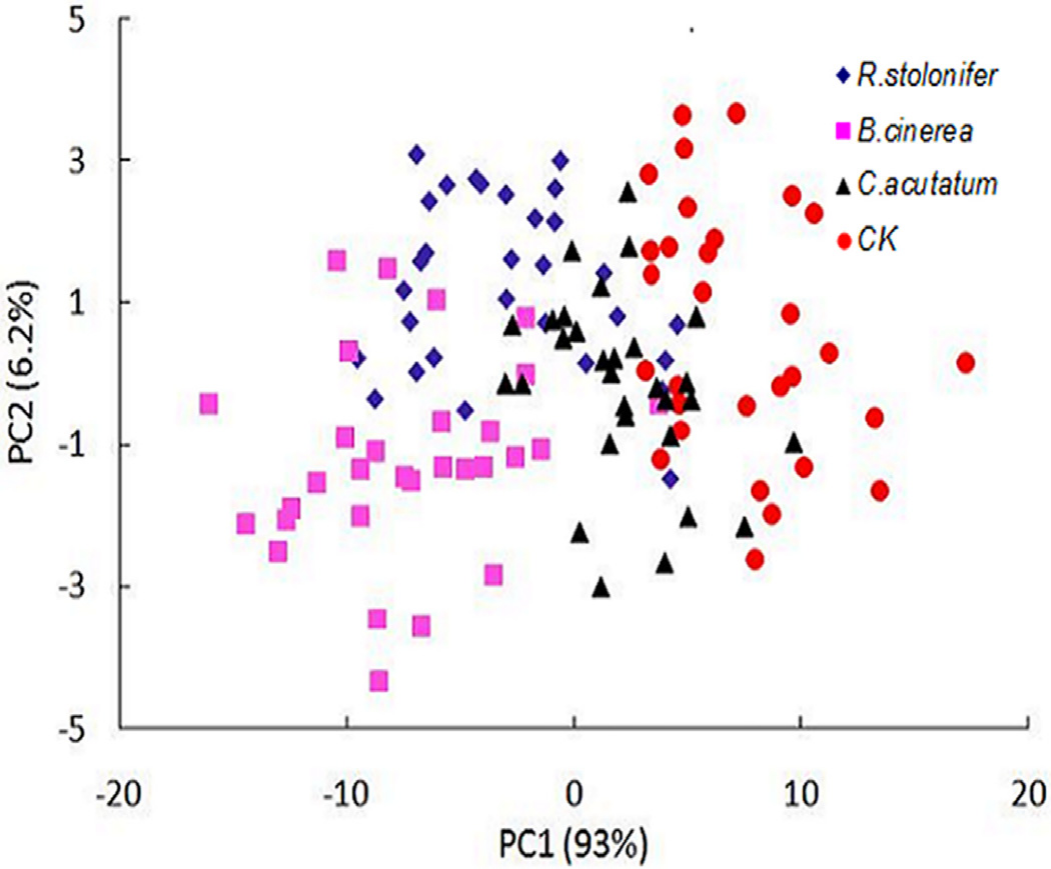
Results of PCA for different fungi at 120 h after inoculation in peach samples.

The statistical analysis performed using PCA on the full spectral ranges (400–1000 nm) was also used to discriminate among the three fungal diseases and control group in peach samples at each of eleven growth stages other than 0 h. The results showed the data points of *B*. *cinerea*, *C*. *acutatum* and *R*. *stolonifer* overlapped on the growth stages from 12 h to 132 h, the distinguishing performance was poor. Fig 7 showed the best result at 120 h during the storage. Part of the data points overlapped among the four groups, the distinguishing effectiveness between control group and other fungi groups was relatively better. These three diseases caused browning and softened on the peach fruits, and there were no significant differences during the storage. The reason was that the peaches only showed, in the early time of decay, the symptoms of browning and softening. There were not apparent difference among peaches infected by different fungi, as same as fungi grew on the plates.

Discrimination accuracy among the four groups at the culture time of 120 h by PLS-DA were 70–90%. The peaches in control group can easy be discriminated from infected peaches with the accuracy of 90%. Therefore, the two-class (‘decay peaches from *B*. *cinerea*, *C*. *acutatum* and *R*. *stoloifenr*’ and ‘non-decay peaches from control group’) classification performed better using by PLS-DA. The model can achieve 100% accuracy between decay and non-decay peaches for the calibration set, while 85% of the validation samples were correctly classified.

### 8. Discussion

In recent years, with the development of various diagnostic or screening technologies working on multidisciplinary platform, the rapid and nondestructive methods for food microorganism detection and identification become more and more mature [32–33]. Hyperspectral image technology was developed in an interdisciplinary effort, such as chemometrics, optics and so on, to advance the method for rapid, non-destructive detection of fungi. Here, we showed the application of hyperspectral image for simulation and discrimination of three kinds of fungal colonies on agar plates. The principle of hyperspectral imaging non-destructive approaches is based on the change of sample structure accompanying the variation of sample mechanical and optical properties [34–35]. So the hyperspectral data is related to both the fungal chemical and physical structural properties, including chemical composition, metabolic products, color, shape, size and so on.

The growth and colony morphology of three fungi at present have been well studied [36–38], those researches show that the colony morphology was easy affected by the culture environment of fungi growth. When the temperature, humidity or culture medium change, several strains will revealed differences in their hyperspectral images, however the influence of the spectrums are insignificant. This research is adopted a fixed cultivation mode, and it makes hyperspectral data were highly reproducible for three fungi, the fixed cultivation mode was also described by Del Fiore et al. [11] and Yao et al. [12].

Among the three fitting methods, the wave crest generated the most matching model with high values of R^2^_c_ and R^2^_p_, and lower values of RMSE and SSE. The model used by wave crest not only reduces the amount of data, but also enhance the accuracy of the model, which are consistent with the effect of optimal wavelengths [29,3]. Some experiment used full spectral range to establish model, then according to some algorithm, the optimal wavelengths were selected. Liet al. [21] indicated that the peaks and valleys always be the dominant optimal wavelengths, so in this study, the wave crest can be regarded as the optimal wavelength, and these are the explanation of the best matching model of growth fitting which use the wave crest.

Through the comparison of fungi growth between pure culture and peach samples, the results of these two experiments were quite different. On the one hand, the fungi inoculated on the plates after a short time reached the lag phase. For the peach samples, it will take a long time to reach the lag phase. So the detection time for peach samples was much longer. What’s more, the CFU of 0 h was less than the actual inoculated concentration, this result was also described by Siripatrawan [27]. Banada [39] and Enos-Berlage [40] reported the effect of composition of the growth medium on the bacteria growth, they found that the variated phase can alter the expression of certain cell surface–associated components, like membrane proteins, exopolysaccharides and lipopolysaccharides. Furthermore, for all tested bacterial, the signatures were different from the different media. The environmental conditions can indirectly affect colony morphology [41]. This was also the reason why it was hard to see the mycelium on the peaches during the detection. On the other hand, the S-shaped growth curve can be clearly found for fungi in the plates, the curve of their growth in the peach samples didn’t reach the stationary phase. This was mainly because fungi surviving in peaches tissue were subject to different surroundings such as acid stress, nutrient composition, which was unlike the growth in the plate [42–43]. Hence, in the process of fruit decay, despite the total numbers of colonies were increased with the enlarging decay area, the fungi still stayed at the exponential phase after 132h of inoculation.

Hyperspectral image system was successfully used for discrimination of fungi species in 36 h (pure cultures). Recently, a Bacterial Rapid Detection using Optical scattering Technology (BARDOT) for microorganism identification was developed by Purdue University [44–46], which is an optical laser sensor-based method, and it can detect microorganism at 7–8 h after inoculation. Compared with the two microorganism detection methods, BARODT need to build a huge image database to ensure the recognition accuracy. What’s more the image is the only standard for BARODT to identification the bacterial; therefore it is easy to affect the recognition result. And the hyperspectral image system with the spectroscopic measurement can correlate the peaks with specific chemical bonds, so it would have highly specific and more reliable results.

## Conclusions

This study aimed to develop a method for modeling the early growth of *B*. *cinerea*, *C*. *acutatum*, and *R*. *stolonifer*, and to discriminate between the three fungal species using hyperspectral imaging. Three methods were used to extract characteristic information of hyperspectral imaging in order to model the growth, the results showed that the R^2^ of growth fitting model for testing datasets of three fungi are 0.7223 to 0.9914, and the correlation coefficient of prediction accuracy between spectral values and colony forming units are 0.887–0.957. Among the three methods, the method of using the wave crest at 716 nm reduced the high spectral dimensionality and achieved with highestR^2^and lowest root mean square error of validation. In addition, fungal species can be distinguished by PCA and PLSDA with the spectral information covering the full wavelength range, and the discrimination accuracy among control, *B*.*cinerea*, *R*. *stolonifer* and *C*. *acutatum* were 97.5% at the culture time of 36 h. What’s more, the hyperspectral imaging system can be used on real food, the R^2^s of growth simulation models for the three fungi in peach were 0.9292–0.9927 and 0.7382–0.8594 in calibration and test datasets, respectively, and the average discrimination accuracy is 75.75% among four groups. The results indicated that hyperspectral imaging has the potential to be a new microbiological modeling technique in the future.

## Supporting Information

S1 Fig Fitting the growth curve of fungi by matlab. (DOCX)

S1 Dataset The original and minimal spectral values of *B*. *cinerea*. (XLSX)

S2 Dataset The original and minimal spectral values of *C*. *acutatum*. (XLSX)

S3 Dataset The original and minimal spectral values of *R*. *stolonifer*. (XLSX)
