# Supplementary material for: Growth Simulation and Discrimination of Botrytis cinerea, Rhizopus stolonifer and Colletotrichum acutatum Using Hyperspectral Reflectance Imaging
Source: PLoS One. 2015 Dec 7;10(12):e0143400. doi: 10.1371/journal.pone.0143400 (PMC4671615; doi:10.1371/journal.pone.0143400)
Supplement: S1 Fig — (DOCX) [file pone.0143400.s001.docx]

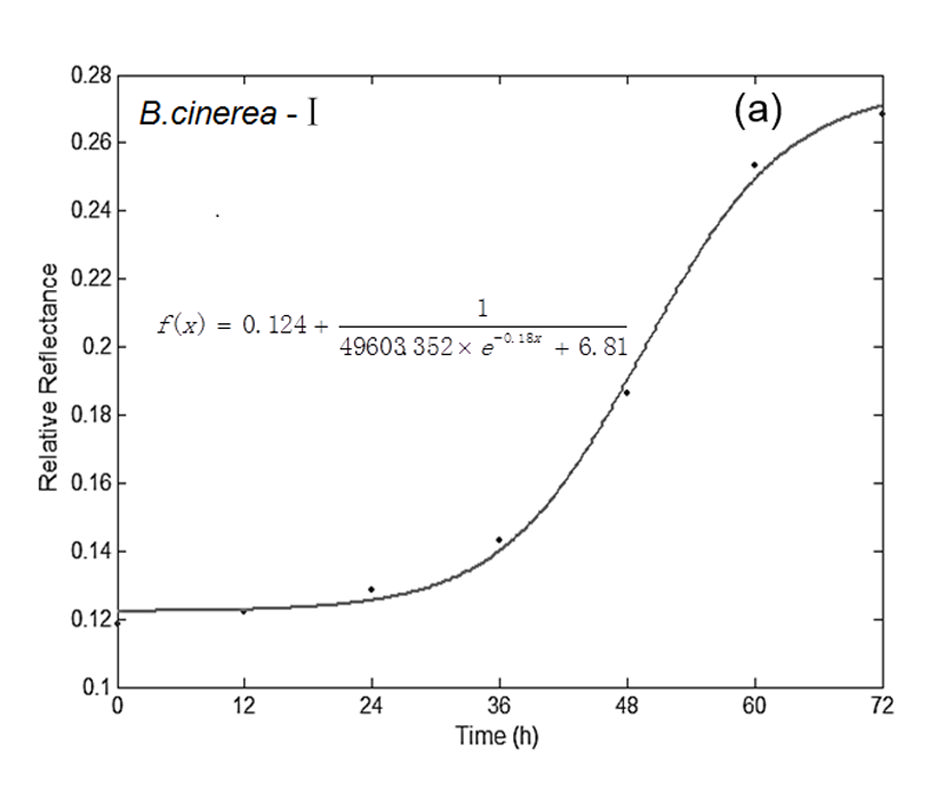

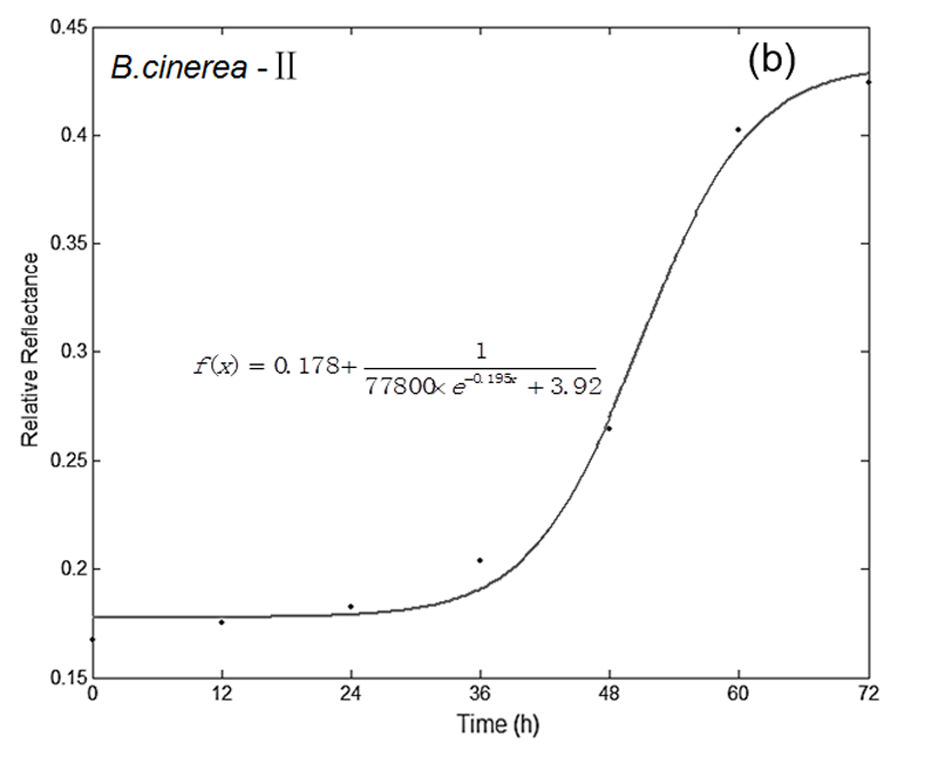

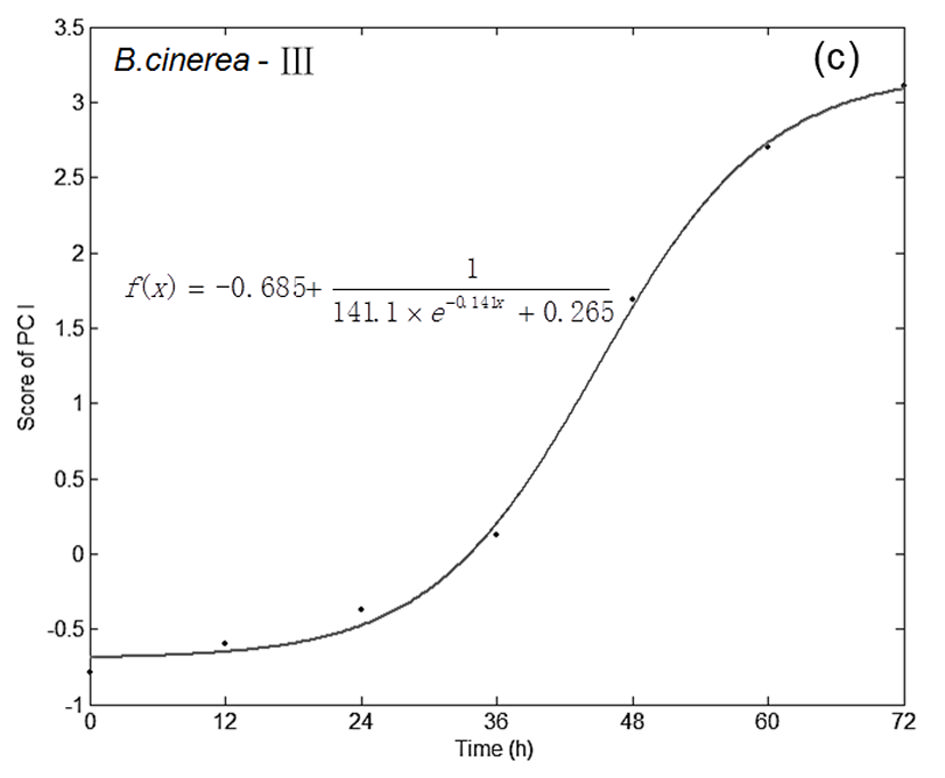

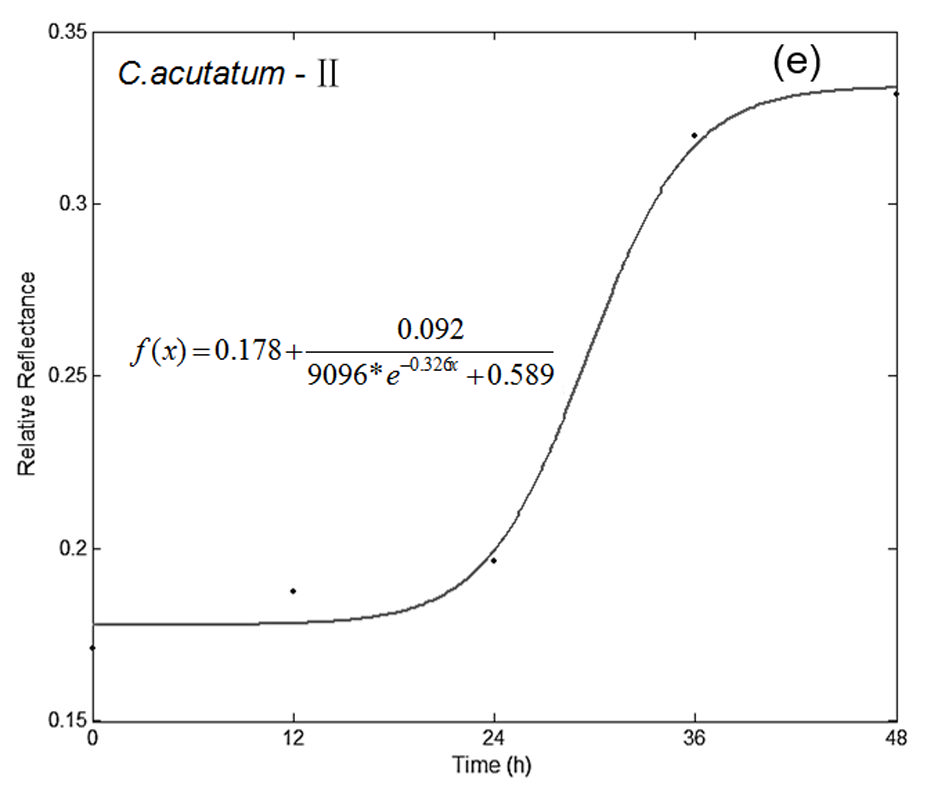


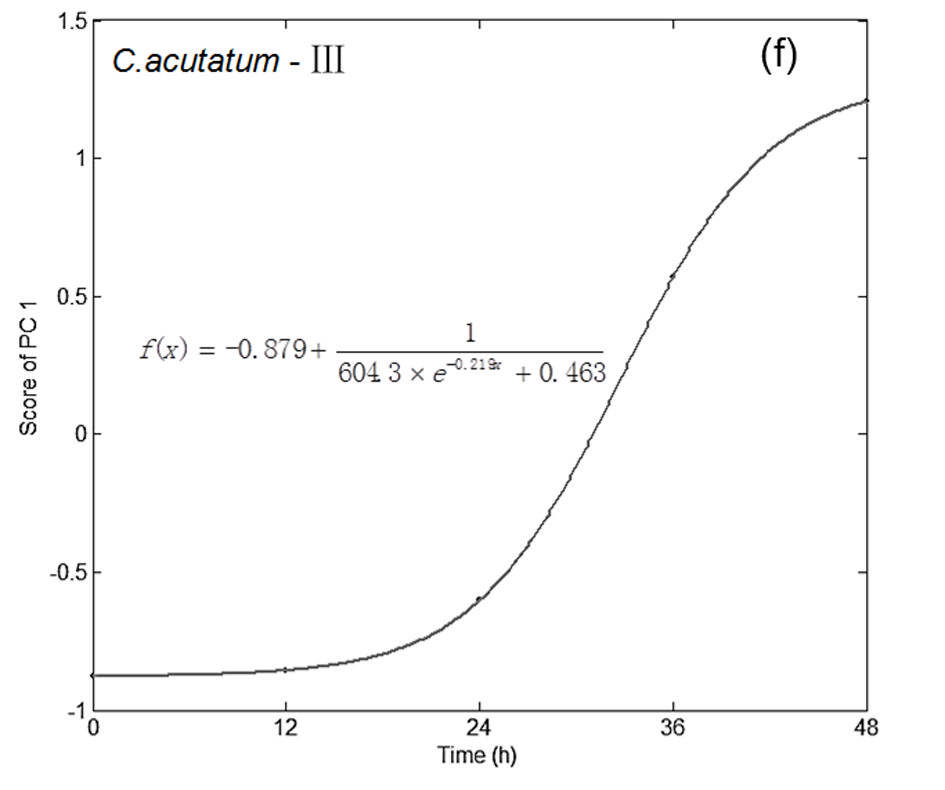


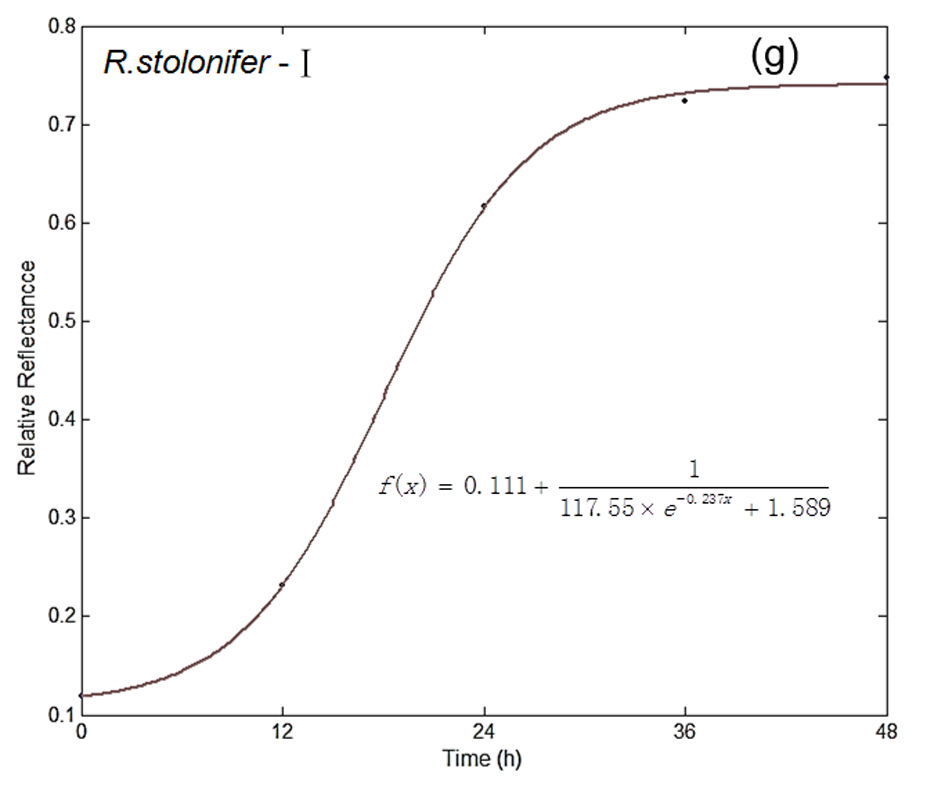


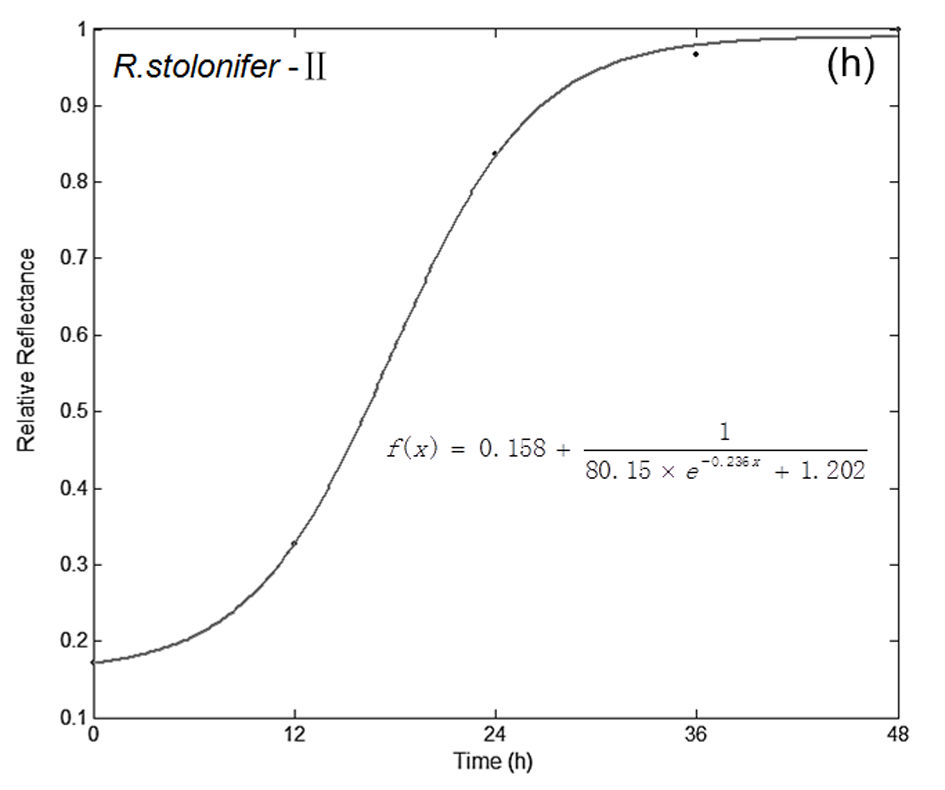

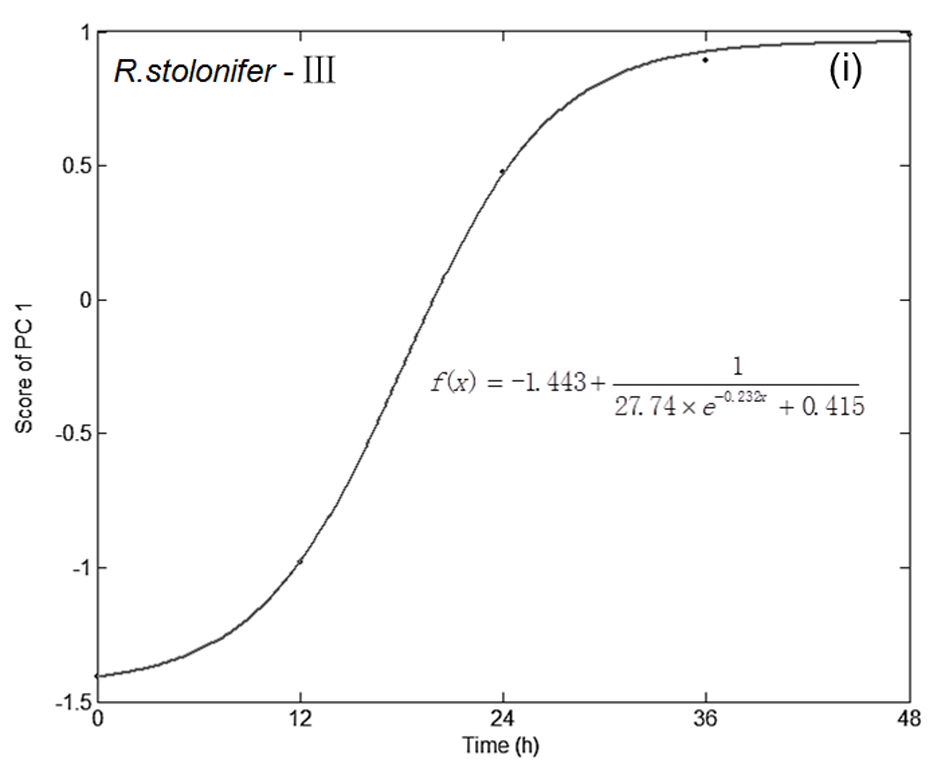


**S1 Fig. Fitting the growth curve of fungi by matlab** (I: using method one of the averages of full wavelengths; II: the response value at wave crest of 716 nm; III: the value of principal component score of full wavelength).
